# Phenotyping the knee joint—A critical appraisal of CPAK for alignment classification

**DOI:** 10.1002/jeo2.70409

**Published:** 2025-09-11

**Authors:** Randa Elsheikh, Yuma Onoi, George M. Avram, Heiko Graichen, Michael T. Hirschmann

**Affiliations:** ^1^ University Department of Orthopedic Surgery and Traumatology Kantonsspital Baselland Liestal Switzerland; ^2^ Department of Clinical Research Research Group Michael T. Hirschmann, Regenerative Medicine & Biomechanics, University of Basel Basel Switzerland; ^3^ Department of Personalised Orthopaedics (PersO) at Privatklinik Siloah Bern Switzerland

**Keywords:** alignment, CPAK, digital twins, knee, knee arthroplasty, phenotypes

**Abbreviations**
aHKA: arithmetic hip‐knee‐ankle angle
AI: artificial intelligence
CPAK: coronal plane alignment of the knee
CT: computed tomography
DTGSA: distal tibial ground surface angle
FJS‐12: Forgotten Joint Score‐12
FMA: femoral mechanical angle
HKA: hip‐knee‐ankle angle
JLCA: joint line convergence angle
JLO: joint line obliquity
KOOS‐12: Knee Injury and Osteoarthritis Outcome Score‐12
LLR: long‐leg radiographs
PTS: posterior tibial slope
TKA: total knee arthroplasty
TMA: tibial mechanical angle

## INTRODUCTION

The last decade has witnessed the intensification of the pursuit of personalisation in total knee arthroplasty (TKA), driven by the mounting evidence that a one‐size‐fits‐all approach to alignment technique might not yield optimal results for every patient [19]. This has led to the questioning of the mechanical alignment technique, which aims at achieving a neutral alignment without properly considering the anatomical variability of the knee. Consequently, several alignment techniques have been developed, all aiming to tailor surgical technique to an individual's native knee morphology and biomechanics [44, 45], although current evidence remains inconclusive as to which approach yields the best outcomes [26]. Clearly, this evolution was accompanied by the need for tools that might aid surgeons in stratifying patients into distinct anatomical or functional categories. Among these tools, the coronal plane alignment of the knee (CPAK) classification has attracted considerable attention, largely owing to its simplified approach and its practical compatibility with routine radiographic assessments [33].

**Figure 1:**
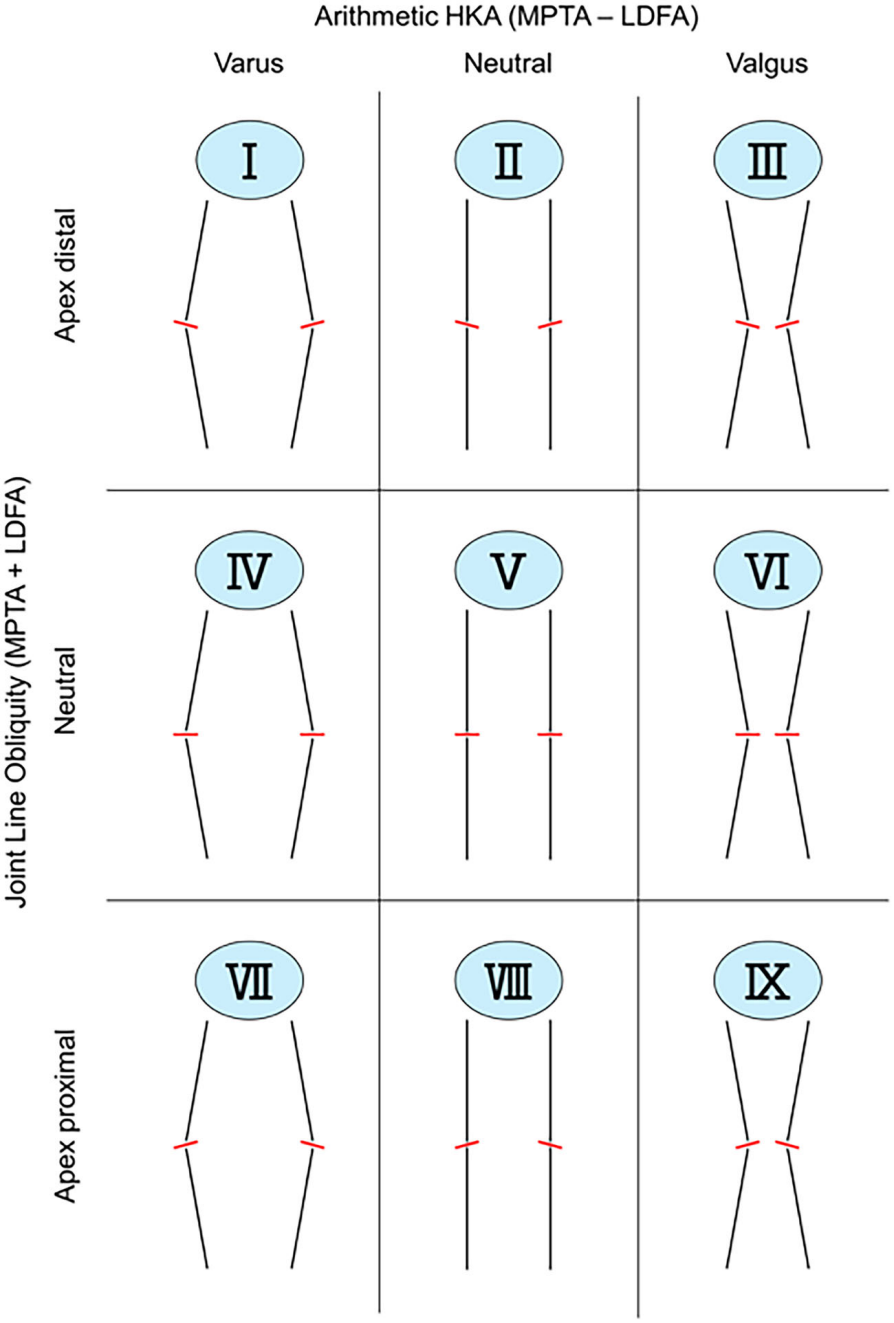
Coronal plane alignment of the knee classification (CPAK) matrix. HKA, hip‐knee‐ankle angle; LDFA, lateral distal femoral angle; MPTA, medial proximal tibial angle.

Originally introduced to support alignment decisions using native coronal plane anatomy, CPAK classifies patients into nine categories based on the arithmetic hip‐knee‐ankle (aHKA) angle and joint line obliquity (JLO) measured on anterior‐posterior knee radiographs [33]. By combining these two values, each knee is classified into a grid of 3 × 3 phenotypes: neutral, varus, or valgus aHKA crossed with apex distal, neutral, or apex proximal JLO (Figure 1). Owing to the ability of the grid‐based approach to offer a simplified yet systematic categorisation of knee alignment while adhering to traditional imaging workflows, CPAK has been frequently described as a method for ‘knee phenotyping’ both in the academic and clinical literature [38, 39, 49]. However, systematic approaches are often based on the notion that all knees are the same and therefore neglect the intrinsic anatomical variability of each joint [14].

Importantly, the use of the term ‘phenotype’ warrants closer scrutiny. Referring to CPAK as a phenotyping system risks overstating its capabilities and oversimplifying a highly complex anatomical and functional construct. In fact, while little variability is observed in mean coronal alignment, substantial inter‐individual variation is detected even in native knees, which makes the classification of coronal alignment into neutral, valgus and varus overly simplistic and rather limiting [14, 15, 19]. Phenotyping, particularly in the context of knee arthroplasty, requires a comprehensive understanding of patient‐specific factors, including three‐dimensional bone morphology and dynamic kinematics. In contrast, CPAK offers a static, two‐dimensional alignment based solely on coronal plane geometry, while ignoring axial and sagittal planes [29].

This editorial explores why CPAK, despite its clinical value, does not fulfil the multidimensional requirements for true phenotyping in knee alignment strategies.

## WHAT IS PHENOTYPING?

The term *phenotype*, originating from the Greek words *phanein* (‘to show’) and *typos* (‘form’ or ‘type’) [24], has become increasingly popular in orthopaedic discourse, as the efforts to pursue a more personalised patient approach intensify. Despite being part of biologists' everyday vocabulary, concepts adopted from biology to surgical practice are often misunderstood or misused.

Originally, a phenotype refers to observable characteristics of an individual, shaped by both genetic predisposition and environmental influences [48]. Translated to orthopaedics, as firstly defined by Hirschmann [17], a knee phenotype should encompass a comprehensive multiplanar assessment of individual morphology, alignment, laxity and kinematics, where one variable cannot be considered without the other.

## LIMITATIONS OF CPAK FOR TRUE KNEE PHENOTYPING

### Anatomical and biomechanical oversimplification

The introduction of the concept of constitutional varus by Bellemans et al. [1] highlighted the considerable variation in native knee alignment. Subsequently, different classification systems have attempted to understand and categorise knee variability as phenotypes based on their coronal alignment characteristics [15, 30, 34, 35]. Based on the same concept, the CPAK classification was developed, categorising patients into nine different phenotypes, using two radiographic parameters: the aHKA angle and JLO, both measured in the coronal plane on long‐leg radiographs (LLR) [33]. Although it incorporates JLO into alignment planning, this system does not account for joint space narrowing or tibiofemoral subluxation and overlooks key factors such as the joint line convergence angle (JLCA), which, together with the HKA angle, influences knee joint line obliquity [23, 36]. Expectedly, CPAK JLO appears to correctly predict true knee apex position only in less than half of the patients [42]. From a biomechanical perspective, there is no such joint line, as this is a 2D description of a 3D phenomenon. In fact, in every knee, there are multiple joint planes dependent on the tibiofemoral contact and the flexion angle of the knee. Therefore, this projected joint line is heavily influenced by loss of extension or hyperextension. Other simplifications are that CPAK neglects the soft tissue variability and fails to account for the distal tibial ground surface angle (DTGSA), hence neglecting the ankle position [37, 42].

When attempting to define coronal alignment, it is important to understand that bony alignment only represents the ‘static’ aspect of this plane. Joint laxity, cartilage wear, and knee kinematics are equally important and intrinsic factors that need to be considered for a comprehensive and dynamic assessment of the knee [16]. Importantly, as it has been previously demonstrated, static alignment cannot predict dynamic alignment [12]. Even within the normality values of bony alignment, inter‐individual variability in joint laxity exists [11, 16]. This underscores the need for independent dynamic knee assessment, particularly owing to the strong correlation of dynamic parameters with TKA outcomes [47].

### Two‐dimensional limitations

Even within static assessment, things have changed. While CPAK focuses on two‐dimensional evaluation, being restricted to the coronal plane, it fails to account for the sagittal and rotational alignment [46]. As opposed to what was stated by Ziegenhorn et al. [50], who claimed the presence of an association between coronal alignment and femoral torsion, Corbett et al. found little correlation between CPAK phenotypes and three‐dimensional alignment [5]. This is also confirmed by recent reports, which show no or only a weak correlation between coronal and rotational alignment parameters [13, 29], emphasising the need for a three‐dimensional evaluation of the knee joint.

Axial alignment is known to affect patellofemoral tracking and soft tissue balance, with malrotation being a recognised cause of postoperative pain, instability, and knee stiffness, often warranting revision [40]. Similarly, sagittal alignment, often termed ‘the forgotten plane’, encompasses parameters such as posterior tibial slope (PTS), which are essential for normal flexion mechanics and directly influence ligament tension, joint stability, and implant kinematics. Additionally, when establishing adequate knee joint balance, the sagittal plane is instrumental in assessing fixed flexion contractures and hyperextension, commonly seen in female patients [41]. Importantly, when evaluating alignment comparability in both legs, significant asymmetry is observed in the coronal plane but also in axial parameters such as tibial torsion and femoral rotation, which are completely unaccounted for in CPAK's framework [43].

Long‐leg radiographs, which form the basis for the CPAK classification, cannot fully capture these planes. In addition, unlike computed tomography (CT), which can accurately reconstruct individual knee morphology, LLRs cannot properly assess key features for true phenotyping, like trochlear groove depth and joint surface wear. Even during the assessment of coronal limb alignment, LLRs tend to underestimate the degree of constitutional varus and JLO when compared to CT scans [25]. Based on the strong correlation between LLRs and CT scanograms [7] and the limitations of the former, the integration of a three‐dimensional assessment in knee phenotyping becomes essential [18].

### Population and gender variability

Besides the lack of three‐dimensionality, the CPAK system suffers from the imposition of arbitrary categorical thresholds on what are inherently continuous variables. These rigid cut‐offs, such as ±2° for aHKA and ±3° for JLO [33], carry the risk of misclassifying patients with nearly identical alignment characteristics. Notably, these thresholds do not account for population variability in native knee alignment, making the system poorly adaptable across different patient groups. This is reflected by the majority of knees being clustered into five CPAK categories, namely I, II, III, IV and VII [24]. An analysis of the lower limb alignment of the Chinese population shows that 92.9% of the knees fall into I–IV CPAK categories, with more than half being classified as type I. In contrast, when applying the functional knee phenotypes classification [15], 140 phenotypes are observed for the same cohort, suggesting the inability of the CPAK classification to fully capture the spectrum of alignment variation in some populations [31]. Similarly, analysis of lower limb alignment in Japanese patients undergoing restricted kinematic alignment TKA highlights the presence of ethnical variability in knee phenotypes, as well as improved femoral varus detection when using the functional knee phenotype classification, underscoring the potential shortcomings of the CPAK classification when applied to patient‐specific alignment concepts [27]. Additionally, gender‐based analyses of knee morphology indicate that, while males and females are predominantly distributed across two and three CPAK types, respectively, more than ten distinct functional knee phenotypes can be identified for each gender within the same cohort [21]. This is corroborated by a recent study showing that as many as 17 phenotypes can be observed for each gender using the functional knee phenotype classification [3].

### Clinical outcomes and misclassification risk

Finally, while some reports suggest that frequent changes in the CPAK classification after TKA do not significantly affect clinical outcomes [2, 4], findings by Konishi et al. challenge this notion. Their study demonstrated that postoperative CPAK changes are associated with worse patient‐reported outcomes, specifically lower Knee Injury and Osteoarthritis Outcome Score‐12 (KOOS‐12) and Forgotten Joint Score‐12 (FJS‐12) [28]. Although these findings highlight the potential clinical relevance of preserving or restoring pre‐arthritic CPAK types, it remains uncertain whether CPAK can reliably serve this purpose. A recent study by Loddo et al. suggests that the CPAK classification, while useful in describing overall alignment phenotypes, falls short in accurately capturing or addressing these segmental deformities [32]. This inability to accurately detect or correct extra‐articular deformities underscores a key limitation in achieving true restoration of the native pre‐arthritic knee phenotype.

## TOWARDS A MORE COMPREHENSIVE PHENOTYPE ASSESSMENT

### Evolving concepts in phenotype‐based knee classification

Hirschmann et al. introduced a more detailed framework for knee alignment classification, intending to capture the constitutional alignment patterns of the femur and tibia relative to the mechanical axis in both healthy and osteoarthritic knees [13, 17]. Often referred to as ‘functional knee phenotype’, this system goes beyond traditional alignment concepts by combining static alignment and functional alignment. The original framework classifies knees based on combinations of three independent angular parameters: the femoral mechanical angle (FMA), the tibial mechanical angle (TMA), and HKA, representing the limb phenotype. Each parameter is stratified into five subcategories (severe varus, varus, neutral, valgus, severe valgus), resulting in a matrix of 125 functional phenotypes. These combinations capture the full spectrum of native coronal lower limb alignment, including cases in which the same limb phenotype may result from different femoral and tibial phenotypes, and vice versa [15, 17]. This disaggregation is critical and represents one key distinction from the CPAK classification. By collapsing native alignment into a two‐variable grid, the CPAK classification fails to acknowledge that two limbs with identical mechanical axes may fundamentally have different underlying bone morphology [8], which has major implications for personalised alignment in TKA. Importantly, this classification is based on three‐dimensional CT imaging [15], allowing accurate characterisation of bone morphology in the coronal plane while preserving spatial anatomical relationships, a level of anatomical accuracy that 2D systems are inherently unable to offer [18].

More recently, the three‐compartment phenotype concept was proposed, which introduces an additional layer of stratification by identifying potential mismatches between distal femoral, posterior femoral, and tibial joint lines [13]. This 3D‐based approach addresses the shortcomings of purely coronal classification systems and represents a forward step toward a more individualised framework for TKA planning. However, even 3D concepts are insufficient in isolation; without accounting for ligamentous balance, surgical correction based on phenotype may not restore the patient's native knee behaviour.

### The need for a dynamic assessment

Recognising this gap, a 3D scoring system was introduced to assess laxity across different personalised alignment strategies and knee types by quantifying medial and lateral gap differences both in extension and flexion. Physiological laxity was better replicated by gap‐balancing techniques, particularly in varus knees, whereas measured resection techniques restored bone geometry but often struggled to reproduce the natural soft‐tissue envelope [9, 10]. This variability in laxity profiles emphasises the importance of incorporating dynamic assessment in knee phenotype concepts.

### Future directions: AI, digital twins and continuous models

A key limitation of current knee classification systems is the exclusive reliance on categorical variables, which fail to entirely capture the variability of individual knee anatomy and alignment [14]. Emerging technologies like artificial intelligence (AI) and digital twins offer a promising solution by enabling a shift toward continuous phenotyping [6, 20]. Digital twin models integrate detailed patient‐specific data, including imaging, biomechanics, and clinical parameters, to create a dynamic, individualised representation of the knee [6]. AI can process and analyse these high‐dimensional datasets, identifying subtle variations and relationships that are otherwise missed by categorical approaches. The integration of these technologies in knee phenotyping will allow for more precise surgical planning and personalisation, eventually improving individualised care [14].

### The importance of phenotype terminology

As evidenced, the CPAK classification does not align with the functional knee phenotype classification, which incorporates crucial elements like knee kinematics, three‐dimensionality, and ligamentous behaviour [22]. This discrepancy suggests that relying exclusively on CPAK for TKA planning may not fully capture the complexity of knee biomechanics and falls short of accurately phenotyping knee alignment.

Clear and precise terminology is essential to advancing research, refining classification systems, and ultimately improving personalised patient care. Therefore, the term ‘phenotype’ should be used cautiously in orthopaedic discourse. CPAK is better described as ‘coronal plane alignment classification’ or ‘coronal plane categorisation,’ terms that more accurately convey its methodological boundaries. Refining such terminology promotes clearer clinical communication, enhances research rigour, and encourages the development of truly multidimensional classification systems that can better serve the evolving goals of personalised medicine in TKA.

Indeed, biology teaches us that the definition of phenotype should strike a balance: it should be neither overly narrow nor overly broad but detailed enough to capture clinically relevant variations.

## AUTHOR CONTRIBUTIONS

*Conceptualisation*: Michael T. Hirschmann. *Methodology*: Michael T. Hirschmann. *Writing—original draft preparation*: Randa Elsheikh and Yuma Onoi. *Writing—review and editing*: Randa Elsheikh, Yuma Onoi, George M. Avram, and Heiko Graichen. Supervision: Michael T. Hirschmann. All authors have read and agreed to the published version of the manuscript.

## CONFLICT OF INTEREST STATEMENT

The authors declare no conflicts of interest.

## ETHICS STATEMENT

Not applicable.
